# Altered Composition of Microbiota in Women with Ovarian Endometrioma: Microbiome Analyses of Extracellular Vesicles in the Peritoneal Fluid

**DOI:** 10.3390/ijms22094608

**Published:** 2021-04-27

**Authors:** Sa-Ra Lee, Jae-Chul Lee, Sung-Hoon Kim, Young-Sang Oh, Hee-Dong Chae, Hochan Seo, Chil-Sung Kang, Tae-Seop Shin

**Affiliations:** 1 Department of Obstetrics and Gynecology, Asan Medical Center, University of Ulsan College of Medicine, Seoul 05505, Korea; leesr@amc.seoul.kr (S.-R.L.); beas100@hanmail.net (J.-C.L.); soccur@hanmail.net (Y.-S.O.); hdchae@amc.seoul.kr (H.-D.C.); 2 MD Healthcare Inc., Seoul 121-270, Korea; hcseo@mdhc.kr (H.S.); cskang@mdhc.kr (C.-S.K.); tsshin@mdhc.kr (T.-S.S.)

**Keywords:** endometriosis, extracellular vesicles, microbiome, 16S rDNA

## Abstract

Human microbiota refers to living microorganisms which colonize our body and crucially contribute to the metabolism of nutrients and various physiologic functions. According to recently accumulated evidence, human microbiota dysbiosis in the genital tract or pelvic cavity could be involved in the pathogenesis and/or pathophysiology of endometriosis. We aimed to investigate whether the composition of microbiome is altered in the peritoneal fluid in women with endometriosis. We recruited 45 women with histological evidence of ovarian endometrioma and 45 surgical controls without endometriosis. Following the isolation of extracellular vesicles from peritoneal fluid samples from women with and without endometriosis, bacterial genomic DNA was sequenced using next-generation sequencing of the 16S rDNA V3–V4 regions. Diversity analysis showed significant differences in the microbial community at phylum, class, order, family, and genus levels between the two groups. The abundance of *Acinetobacter*, *Pseudomonas*, *Streptococcus*, and *Enhydrobacter* significantly increased while the abundance of *Propionibacterium*, *Actinomyces*, and *Rothia* significantly decreased in the endometriosis group compared with those in the control group (*p* < 0.05). These findings strongly suggest that microbiome composition is altered in the peritoneal environment in women with endometriosis. Further studies are necessary to verify whether dysbiosis itself can cause establishment and/or progression of endometriosis.

## 1. Introduction

Endometriosis is the growth of the endometrial tissue at extra-uterine sites. The tissue is most commonly implanted over and under visceral and peritoneal surfaces within the female pelvis but it can also be found in the connective tissue of the extrapelvic region [1,2,3]. Endometriosis may cause severe dysmenorrhea, pelvic pain, and infertility, and can seriously deteriorate fertility and quality of life in women. The prevalence of endometriosis has been reported to be as high as 10%–15% in women of reproductive age, and its incidence rate is increasing each year [4,5,6]. Despite a long history of basic and clinical research into endometriosis, the mechanism underlying this pathology remains unclear [7,8,9]. Although Sampson’s theory that implantation of endometrial tissues follows retrograde menstruation seems to be the most plausible hypothesis, it needs to be determined as to why only a certain group of women suffer from the disease.

Human microbiota comprises living microorganisms which colonize our body and play a crucial role in the metabolism of nutrients and various physiological functions. Specifically, the gut microbiota synthesize vitamins B12 and K, provides intestinal mucosal integrity, promotes angiogenesis and epithelial repair, and modulates immune functions [10,11]. Recently accumulated evidence shows that the disruption of gut microbiota may lead to development and progression of diverse diseases, such as inflammatory bowel diseases, arthritis, psoriasis, neuropsychiatric diseases, and even malignancies [12,13]. Given that endometriosis is a chronic inflammatory disease that might be caused by altered immune functions and increased angiogenesis, dysbiosis of human microbiota in the genital tract or pelvic cavity could be involved in the pathogenesis and/or pathophysiology of endometriosis. Indeed, several studies have shown an increased bacterial colonization of menstrual blood and endometrial tissue in women with endometriosis compared with that in control subjects [14,15,16,17]. A study on rhesus monkeys demonstrated that altered composition of the intestinal microflora and intestinal inflammation are associated with endometriosis [18]. A recent study compared the vaginal, cervical, and gut microbiota between women with and without advanced stage endometriosis and showed that some potentially pathogenic species were increased in the cervical and stool microbiome in women with advanced stage endometriosis [19].

Because endometriosis develops in the pelvic cavity in most cases, we focused on the peritoneal fluid (PF) to investigate the possible role of microbiota in endometriosis. Considering that the pelvic cavity is very close to the intestinal tract as well as to the lower genital tract, it is possible that gut microbiota-derived extracellular vesicles (EVs) traverse the intestinal barrier and directly affect the peritoneal environment, because the diameter of these nanovesicles ranges from 20 to 400 nm [20]. Therefore, we analyzed the EVs in the PF samples from women with advanced stage endometriosis and controls (women without the disease) and investigated whether the composition of microbiota is altered in women with endometriosis using next-generation sequencing (NGS) of the 16S rDNA V3–V4 regions.

## 2. Results

Significant differences in the microbial community were observed in the phylum, class, order, family, and genus levels between the two groups, showing that the microbiome composition is altered in the peritoneal environment in women with advanced stage endometriosis.

### 2.1. Microbiome Analysis of EVs in PF: Alpha Diversity

**Figure 1:**
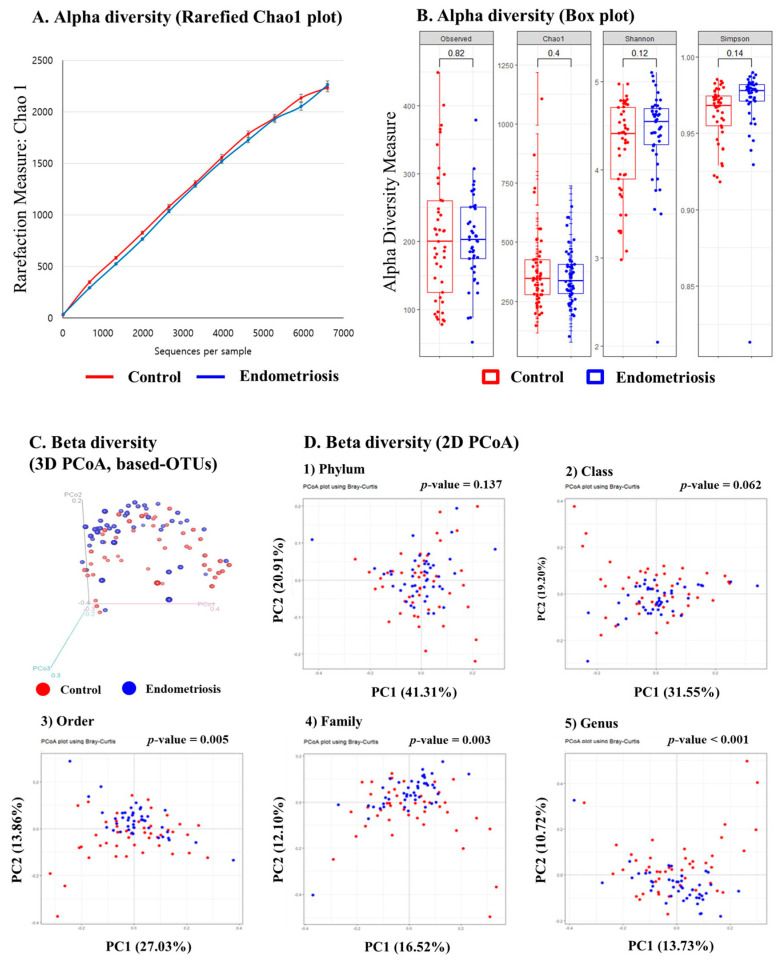
Composition of peritoneal fluid microbiota-derived extracellular vesicles and principal component analysis (PCoA) in the control (**red**) and endometriosis (**blue**). groups (**A**) Alpha diversity measured using the Rarefied Chao1 plot; (**B**) Alpha diversity measured using the Observed, Chao1, Shannon, and Simpson box plots; (**C**,**D**) Beta diversity analysis was conducted to see the differences in the composition of the microbial community between the control (**red**) and endometriosis (**blue**) groups. The analysis was performed using the Bray–Curtis diversity analysis, and the PCoA plot is shown. Each dot represents one sample. The percentages on the axes indicate the contribution rate of each principal component. (**A**) Beta diversity in 3D PCoA based operational taxonomic units; (**B**) Beta diversity in 2D PCoA by phylum, class, order, family, and genus levels.

We investigated whether different bacterial components would be harbored in EVs in the PF samples from women with and without endometriosis. As shown in Figure 1A,B, the observed (*p* = 0.82), Chao1 (*p* = 0.4), Shannon (*p* = 0.12), and Simpson (*p* = 0.14) diversity analysis did not show any obvious differences in species richness between the two groups.

### 2.2. Microbiome Analysis of EVs in PF: Beta Diversity

To further investigate the distribution of microbiota in the PF microbiota-derived EVs, a comprehensive microbiome analysis was performed using NGS (Figure 1C,D). We examined the distribution of the microbial community in PF microbiota-derived EVs in both the control and endometriosis groups. Beta diversity analysis indicated a significant difference in the microbial community between the control and endometriosis groups in 3D principal coordinate analysis (PCoA)-based operational taxonomic units (OTUs) (Figure 1C, *p* < 0.001). Bray–Curtis beta diversity analysis indicated significant differences in the microbial community in order (*p* = 0.005), family (*p* = 0.003), and genus (*p* < 0.001) between the two groups (Figure 1D).

### 2.3. Microbiome Composition of EVs in PF in Women with and without Endometriosis

**Figure 2:**
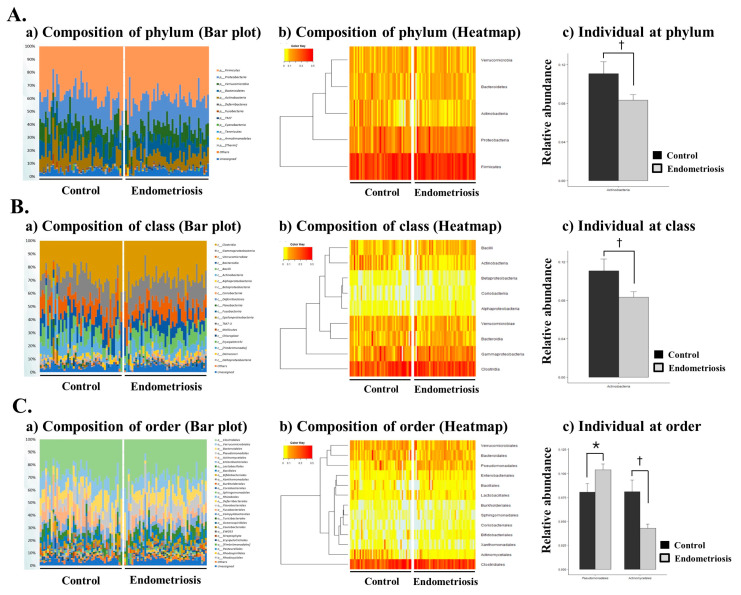
Microbial composition of the peritoneal fluid in the control and endometriosis groups at the phylum, class, and order levels. (**A**) (**a**,**b**) microbiome composition at the phylum level (Bar plot and Heatmap), (**c**) individual microbiome at the phylum level; (**B**) (**a**,**b**) microbiome composition at the class level (Bar plot and Heatmap) (**c**) individual microbiome at the class level, (**C**) (**a**,**b**) microbiome composition at the order level (Bar plot and Heatmap) (**c**) individual microbiome at the order level. † *p* < 0.05, * *p* < 0.05, respectively.

The microbiome of EVs in the PF samples contained various populations of individual bacterial species. Several different types of bacteria were detected in the PF microbiota-derived EVs in both groups, and Actinobacteria were the most frequently observed [Figure 2A(a)]. Microbiome abundance analysis at the phylum level revealed that Actinobacteria, Firmicutes, Proteobacteria, Verrucomicrobia, Bacteroidetes, Deferribacteres, Fusobacteria, Cyanobacteria, Tenericutes, Armatimonadetes, Thermi, Euryarchaeota, Chloroflexi, Spirochaetes, Planctomycetes, Acidobacteria, Gemmatimonadetes, Synergistetes, and Lentisphaerae were the most abundant taxa. There was a significant decrease in Actinobacteria at the phylum level in women with endometriosis compared with the controls [Figure 2A (b and c), *p* < 0.05].

Microbiome abundance analysis at the class level revealed that Clostridia, Gammaproteobacteria, Verrucomicrobiae, Bacteroidia, Bacilli, Actinobacteria, Alphaproteobacteria, Betaproteobacteria, and Coriobacteriia were the most abundant taxa [Figure 2B(a)]. There was a significant decrease in Actinobacteria at the class level in women with endometriosis [Figure 2B (b,c), *p* < 0.05].

**Figure 3:**
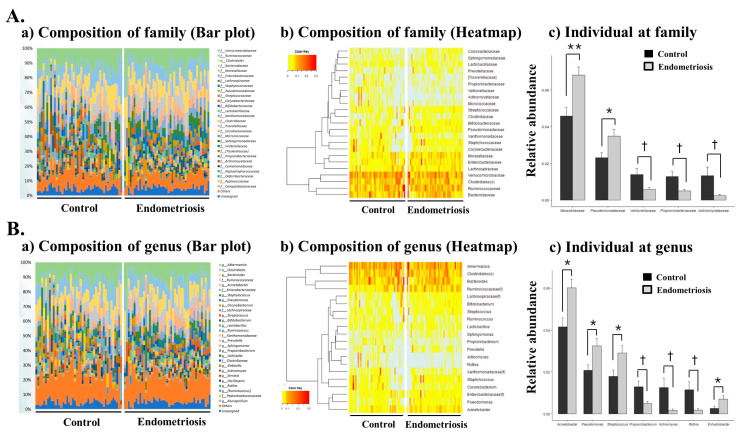
Microbial composition of the peritoneal fluid in the control and endometriosis groups at the family and genus levels. (**A**) (**a**,**b**) microbiome composition at the family level (Bar plot and Heatmap) (**c**) individual microbiome at the family level; (**B**) (**a**,**b**) microbiome composition at the genus level (Bar plot and Heatmap) (**c**) individual microbiome at the genus level. * *p* < 0.05, ** *p* < 0.001, † *p* < 0.05, respectively.

Microbiome abundance analysis at the order level revealed that Clostridiales, Verrucomicrobiales, Bacteroidales, Pseudomonadales, Actinomycetales, Enterobacteriales, Lactobacillales, Bacillales, Bifidobacteriales, Xanthomonadales, Burkholderiales, Coriobacteriales, and Sphingomonadales were the most abundant orders [Figure 2C(a)]. There was a significant decrease in Actinomycetales and a significant increase in Pseudomonadales at the order level in women with endometriosis [Figure 2C (b,c), Table 1, *p* < 0.05]. At the family level, Moraxellaceae was the most abundant, and Pseudomonadales and Moraxellaceae were significantly increased in women with endometriosis (Figure 3A, Table 1, *p* < 0.05 and *p* < 0.001, respectively). However, a sharp decrease of Veillonellaceae, Propionibacteriaceae and Actinomycetaceae abundance was recorded in women with endometriosis (Figure 3A, Table 1, *p* < 0.05).

At the genus level, Acinetobacter was the most abundant taxon, and Acinetobacter, Pseudomonas, Streptococcus, and Enhydrobacter were significantly increased in women with endometriosis. There was a marked decrease in the abundance of Propionibacterium, Actinomyces and Rothia in women with endometriosis (Figure 3B, Table 1, *p* < 0.05, respectively). The microbiota composition of EVs in the PF was significantly different between women with advanced stage endometriosis and without endometriosis (or controls), suggesting that microbiota-derived EVs in the PF might play a role in the pathogenesis and/or pathophysiology of endometriosis.

The microbial composition of the PF was similar between those with endometriosis stage III and IV. We could see significant differences between the two groups only in Enterobacteriaceae (*p* < 0.01) and Propionibacterium (*p* < 0.01) at genus level. When we compared the microbial composition of the PF between the control women with myoma and non-endometriotic ovarian cyst, we could see a significant difference only in Bacteroides (*p* < 0.01) between the two groups at the genus level. To evaluate whether there is any difference of the microbial composition according to age, we divided all of the subjects into three age groups (age less than 30, age of 30 to 40 years, age over 40), and compared the microbial composition among the three groups. There were no significant differences of the microbial composition among the three groups.

## 3. Discussion

EVs are small structures made of bilayered lipid membranes that cannot replicate themselves, and they are released by diverse eukaryotic cells. They are composed of three subpopulations including exosomes, microvesicles, and apoptotic bodies. Because they carry a cargo of proteins, nucleic acids, and lipids, they play a key role in immune function, inflammatory reaction, and disease development, by transporting active molecules to distant sites [21]. Like any eukaryotic cell, bacteria can release EVs of a very small size (below 300 nm) that can modulate host-cell immune responses and other health statuses [22]. It has been suggested that EVs from commensal bacteria may have beneficial effects on the host by enhancing their mucosal tolerance and preventing disease progression, whereas EVs from pathologic bacteria have proinflammatory effects on the host immune cells [22,23]. While gut microbiota are restricted to the intestinal lumen, the secreted EVs can penetrate through the intestinal barriers and enter the systemic circulation, and affect both adjacent and distant organs. Indeed, several studies have shown that gut microbiome-derived EVs play a critical role in the regulation of immune and metabolic functions as key communication messengers between the gut microbes and host, causing inflammatory disease and insulin resistance [24,25].

A recent study has demonstrated that EVs from women with endometriosis carry unique cargo and contribute to disease pathophysiology by influencing inflammation, angiogenesis, and proliferation [26]. Another study has shown that exosomes are present in the PF samples, and that specific proteins in the exosomes are found only in patients with endometriosis, suggesting a role of exosomes in the diagnosis and treatment of endometriosis [27]. Given that dysbiosis of microbiota in the genital tract or peritoneal cavity could lead to the establishment and/or progression of endometriosis [14,15,16,17,18,19], it seems plausible that the composition of microbiota-derived EVs is altered in the PF samples of patients with endometriosis. However, to the best of our knowledge, there has been no study to analyze microbiota-derived EVs in PF from patients with and without endometriosis.

In the present study, we analyzed the EVs in PF samples of women with and without endometriosis and investigated whether the composition of microbiota is altered in women with endometriosis by NGS of the 16S rDNA V3–V4 regions. We identified significant alterations in microbiota composition of EVs in the PF of women with endometriosis compared with that in the controls. Diversity analysis showed significant differences in the microbial community at order, family, and genus levels between the two groups. The abundance of *Acinetobacter*, *Pseudomonas*, *Streptococcus*, and *Enhydrobacter* was significantly increased in the endometriosis group compared with that in the control group, whereas the abundance of *Propionibacterium*, *Actinomyces*, and *Rothia* was obviously decreased in the endometriosis group compared with that in the control group.

It is difficult to identify any microorganism in the peritoneal environment in subjects without any pelvic infectious disease using traditional culture methods. However, a recent study using NGS suggested that the uterine cavity and pelvic environment are not sterile and distinct microbial community is harbored throughout the female reproductive tract from the vaginal canal to the peritoneal fluid [28]. *Acinetobacter*, *Pseudomonas*, and *Comamonadaceae* were the most abundant bacteria within the endometrium, pouch of Douglas, and fallopian tubes [28]. Another NGS study, using microbiome analysis of mid-endometrial samples of hysterectomy specimens, reported that these three bacteria and *Cloacibacterium* are dominant in the uterus [29].

Recently, two studies compared the composition of microbiota between women with and without endometriosis. Ata et al. compared the vaginal, cervical, and gut microbiota between 14 women with advanced stage endometriosis and 14 healthy controls using NGS of the 16S rDNA V3–V4 regions [19]. They found that *Atopobium* was completely absent in the vaginal and cervical microbiota in patients with endometriosis, and *Gardnerella*, *Streptococcus*, *Escherichia*, *Shigella*, and *Ureaplasma* were increased in the cervical microbiota of women with endometriosis. However, *Gardnerella* and *Ureaplasma* are well-known pathogens of bacterial vaginitis and it is not clear whether they are also present in the PF or affect the pathogenesis of endometriosis. Wei et al. also analyzed the samples from vagina, posterior vaginal fornix, cervical mucus, endometrium, and PF from 36 women with endometriosis (16 patients in stages I–II and 20 patients in stages III–IV) and 14 controls using NGS of the 16S rDNA V4–V5 regions [30]. They found that the proportion of *Lactobacillus* was less in the vagina and posterior vaginal fornix in patients with endometriosis compared with that in the controls. They also reported that *Sphingobium sp*. and *Pseudomonas viridiflava* were significantly enriched in the endometrium as well as in the PF in patients with endometriosis and suggested that these two species might play a key role in the pathogenesis of endometriosis [30].

Our data appear to be similar with those reported by Wei et al., who showed that *Acinetobacter* and *Pseudomonas* are significantly enriched in the PF in patients with endometriosis by analyzing peritoneal microbiome [30]. Another recent NGS study showed a different bacterial composition in deep infiltrating endometriosis lesions compared with the control eutopic endometrium, in which *Pseudomonas*, *Enterococcus*, and *Alishewanetta* were more abundant in deep endometriotic lesions [31]. Based on the findings of several studies showing that *Acinetobacter* and *Pseudomonas* are principal members in the endometrial cavity [27,28,29,30], it is possible to assume that increased colony formation by the two bacteria can lead to the establishment of endometriosis in the pelvic cavity through the activation of inflammatory pathways. According to the bacterial contamination hypothesis (14), the altered microbial compositions of the EVs in the peritoneal environment could lead to establishment and progression of endometriosis by triggering the gene expression of a number of target molecules such as cytokines, chemokines, and growth factors through lipopolysaccharide and the toll-like receptor 4 cascade signaling pathway. However, it is also possible that increased colony formation by some bacterial species can be caused by the abnormal peritoneal environment in women with endometriosis, in which immunological dysfunction may have direct effects on bacterial replication. Specifically, Khan et al. suggested that higher prostaglandin E2 levels in the PF of women with endometriosis might cause bacterial replication by immunosuppressive effect [32]. Therefore, further studies are necessary to verify whether dysbiosis itself can cause establishment of endometriosis or whether it is an epiphenomenon accompanied by immunological alteration caused by preexisting endometriosis.

To our knowledge, the present study is the first to focus on the EVs of the PF in endometriosis in terms of microbiota composition. Because the microbiota-derived EVs in the PF may originate not only from the lower genital tract but also from the intestinal tract in the pelvic cavity, the present study is significant in that the EVs were isolated to analyze the microbiota composition in women with and without endometriosis. Moreover, to our knowledge, the present study recruited the largest number of cases (*n* = 45) and controls (n = 45) to compare the microbiota composition in the PF between two groups.

However, the present study has several limitations. First, we could analyze the microbiota composition only in patients with advanced stage endometriosis but could not provide any data during the initial establishment of endometriosis. Therefore, we could not demonstrate that the different microbiota composition was involved in the pathogenesis and/or pathophysiology of endometriosis. Second, the identified genera were the most abundant types present in both groups because the procedures employed in this study were not applicable for detection of rare genera. Thus, it is possible that undetected bacterial genera might be associated with pathogenesis and/or pathophysiology of endometriosis. Third, we could not clarify whether the altered microbiota composition of EVs in PF samples of patients with endometriosis originates from the gut or the lower genital tract. Therefore, further studies are necessary to compare the microbiota composition in lower genital tract as well as in feces between patients with and without endometriosis. Finally, the controls were not healthy controls and had uterine leiomyoma or benign ovarian cysts. Considering that peritoneal microbiota might be also affected by presence of benign gynecological diseases, a further study recruiting healthy controls without disease is necessary to validate the findings of the present study.

## 4. Materials and Methods

### 4.1. Subjects, Collection, and Preparation of PF Samples

Forty-five women with histological evidence of endometriosis and 45 surgical controls without endometriosis were enrolled from Asan Medical Center. PF was obtained from the posterior cul-de-sac or utero-vesical pouch through the laparoscopic cannula in the follicular phase during laparoscopic surgery. The cellular components of the PF were removed by centrifugation at 3500× *g* for 15 min. The PF supernatant was then collected and stored in aliquots at −70 to −80 °C until the analysis. All the patients in the endometriosis group were confirmed as having ovarian endometrioma by histological evaluation, and the stages were classified according to the revised American Society for Reproductive Medicine (r-ASRM) scoring system [33]. All the controls were confirmed as having no endometriotic lesions by laparoscopy and had histological diagnoses of uterine leiomyoma (n = 31) or benign ovarian cyst (n = 14). Women who had taken progestin, oral contraceptives, gonadotropin-releasing hormone agonist, antibiotics, or probiotics within 12 weeks prior to enrollment in this study and those who were diagnosed with inflammatory bowel disease or cancer were excluded. The clinical characteristics of the cases and controls are summarized in Table 2. Written informed consent was obtained from each patient using a consent form. The protocols were approved by the Institutional Review Board for Human Research of Asan Medical Center (approval No. 2014-1165).

### 4.2. Isolation of EV and DNA Extraction

**Figure 4:**
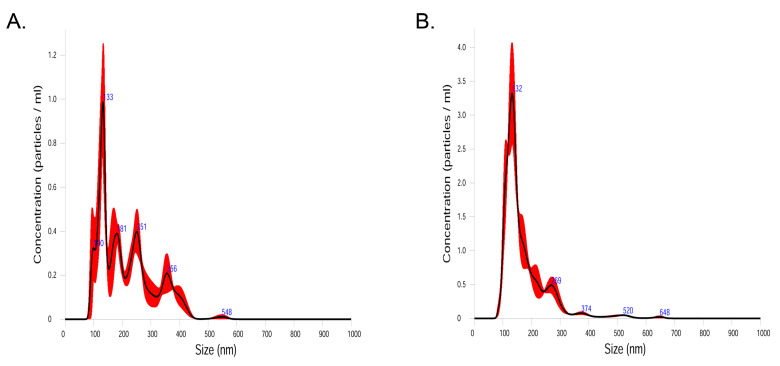
Concentration of particles according to size (nm) in control samples (**A**) and samples from endometriosis group (**B**).

Human PF samples were filtered through a 75 μm cell strainer after being diluted in 10 mL of phosphate-buffered saline for 24 h. To separate EVs from PF samples, EVs in the samples were isolated by centrifugation at 10,000× *g* for 10 min at 4 °C. After centrifugation, the pellet of PF samples contained bacterial cells, and the supernatant of PF samples contained EVs. Bacteria and foreign particles were eliminated by sterilization of the PF sample supernatant by passing it through a 0.22 µm filter. To validate the isolation process of EVs, we analyzed the size of the particles utilizing nanoparticle tracking analysis (NTA). Randomly selected PF samples (2 from controls, and 2 from endometriosis group) were loaded into the NS300 unit chamber (Malvern Panalytical, UK). Each sample was recorded triplicately and underwent Brownian motion in a 1 ml chamber through the 642 nm laser beam at 25 °C. Data analysis was performed on NTA 3.4 build (Nanosight). Capture settings were: Laser Type: Red; Camera Level: 13; Slider Shutter: 1232; Slider Gain: 219; FPS 25.0; Software settings for analysis were: Detection Threshold: 5; Blur: auto; Max Jump Distance: Auto: 9.1–9.9 pix. As shown in Figure 4, the size of the particles was below 300 nm in 80.4% in control samples (mean: 214.1 nm; mode: 127.8 nm) and 94.1% in samples from the endometriosis group (mean: 173.7 nm; mode: 128.0 nm), which strongly supports the validity of isolation processes in the present study.

To extract DNA from bacterial EVs, EVs were boiled at 100 °C for 40 min. To eliminate the remaining floating particles and waste, the supernatant was collected after 30 min of centrifugation at 13,000 rpm at 4 °C. Total DNA was extracted using a DNA isolation kit (PowerSoil^®^ DNA Isolation Kit, MO BIO Laboratories Inc.) in accordance with the manufacturer’s instructions. The DNA extracted from the EVs contained in each sample was quantified using a QIAxpert system (QIAGEN).

### 4.3. PCR Amplification and Pyrosequencing

Bacterial genomic DNA was amplified with 16S_V3_F (5′-TCGTCGGCAGCGTCAGATGTGTATAAGAGACAGCCTACGGGNGGCWGCAG-3′) and 16S_V4_R (5′-GTCTCGTGGGCTCGGAGATGTGTATAAGAGACAGGACTACHVGGGTATCTAATCC-3′) primers targeting the V3–V4 hypervariable regions of the 16S rDNA gene. Libraries were prepared using PCR products according to the MiSeq System guide (Illumina) and quantified using the QIAxpert system. Each amplicon was then quantified, set to an equimolar ratio, pooled, and sequenced with MiSeq according to the manufacturer’s instructions.

### 4.4. Metagenomic Data Analysis of Human PF Samples

Alpha diversity of the samples was measured by determining the observed species, Shannon diversity, Simpson diversity, and the Chao1 indices. The observed species index measures the number of different species per sample, which is defined as “richness.” The Chao1 index is also a qualitative measure of alpha diversity. However, regarding diversity, not only the qualitative amount of species, but also the abundance of the species must be taken into account. The relative abundance of the different species making up the samples’ richness is defined as “evenness”. The Shannon diversity index relates to both, OTUs richness and evenness. Simpson diversity index is a measure of diversity, which considers species richness and the evenness of abundance among the species that are present. In essence, it measures the probability of two individuals randomly selected from an area belonging to the same species. The association between microbial diversity and endometriosis was tested via rarefaction and box plots. Beta diversity analysis represents the extent of similarity between different microbial communities and was calculated based on a PCoA plot. Beta diversity analysis was performed with Bray–Curtis distance matrices and visualized using the PCoA plot. The Kruskal–Wallis test was performed with log normalized data to identify imbalances in abundance only. The significance level was ** *p* < 0.01.

### 4.5. Analysis of Microbial Composition

Raw pyrosequencing reads obtained from the sequencer were filtered according to the barcode and primer sequences using a MiSeq (Illumina). Taxonomic assignment was performed by the profiling program, MDx-Pro ver.1 (MD Healthcare), which selects high-quality sequencing reads with read lengths >300 bp and Phred scores >20 (>99% accuracy of base call). OTUs were clustered using the sequence clustering algorithm, CD–HIT. Subsequently, taxonomy assignment was carried out using UCLUST and QIIME against the 16S rDNA sequence database in Greengenes 8.15.13. [34]. Based on sequence similarities, taxonomic assignment to the genus level was performed on all the 16S rDNA sequences. The bacterial composition at each level was plotted in a stacked bar graph. In cases where clusters could not be assigned at the genus level due to the lack of sequences or redundant sequences in the database, the taxon was assigned at the next highest level, as indicated in parentheses. To avoid potential bias caused by differing sequencing depths, samples with >1000 reads were rarefied to a depth of 1332 reads for subsequent analysis. We also provided basic statistical analysis of the differences between groups, including Student’s *t*-test based on the normalized OTU reads of taxa from the phylum at the genus level. Alpha diversity was calculated by the number of observed OTUs and the Chao 1, Shannon, and Simpson indices. Each alpha diversity value was analyzed with Student’s *t*-test. Beta diversity was calculated with the Bray–Curtis Dissimilarity Index. Each beta diversity value was analyzed using permutational multivariate analysis of variance.

## 5. Conclusions

The microbiota composition of EVs in the PF is significantly different between women with advanced stage endometriosis and without endometriosis (or controls). These data suggest that microbiota-derived EVs in the PF might play a role in the pathogenesis and/or pathophysiology of endometriosis.

## Figures and Tables

**Table 1:** Composition of the microbiota in the control and endometriosis groups at the phylum, class, order, family, and genus levels.

	Control		Endometriosis		*t*-test
Taxon	Mean	SD	Mean	SD	*p*-Value
Phylum					
*Actinobacteria*	0.1107	0.0815	0.0831	0.0394	0.0477
Class					
*Actinobacteria*	0.1004	0.0784	0.0705	0.0368	0.0255
Order					
*Pseudomonadales*	0.0805	0.0606	0.1037	0.0411	0.0387
*Actinomycetales*	0.0811	0.0792	0.0429	0.0284	0.0039
Family					
*Moraxellaceae*	0.0459	0.0306	0.0682	0.0298	0.0008
*Pseudomonadaceae*	0.0230	0.0189	0.0348	0.0244	0.0128
*Veillonellaceae*	0.0139	0.0227	0.0058	0.0069	0.0282
*Propionibacteriaceae*	0.0129	0.0190	0.0049	0.0062	0.0110
*Actinomycetaceae*	0.0133	0.0309	0.0023	0.0058	0.0243
Genus					
*Acinetobacter*	0.0416	0.0289	0.0602	0.0265	0.0022
*Pseudomonas*	0.0208	0.0175	0.0325	0.0235	0.0097
*Propionibacterium*	0.0129	0.0190	0.0049	0.0062	0.0111
*Streptococcus*	0.0179	0.0204	0.0291	0.0231	0.0183
*Rothia*	0.0115	0.0261	0.0018	0.0045	0.0190
*Actinomyces*	0.0126	0.0305	0.0017	0.0043	0.0233
*Enhydrobacter*	0.0026	0.0072	0.0071	0.0114	0.0300

SD, standard deviation.

**Table 2:** Clinical characteristics of patients and controls.

	Endometriosis Group (*n* = 45)	Control Group (*n* = 45)	*p*-Value
Age	36.2 ± 1.3	39.4 ± 1.1	0.06 ^a^
No. of deliveries	0.7 ± 0.1	1.3 ± 0.1	<0.01 ^a^
Body mass index (kg/m^2^) Married women (%) Infertility (%) AFS classification of endometriosis	21.0 ± 0.5 25 (55.6%) 2 (4.4%)	22.2 ± 0.5 32 (71%) 0	0.18 ^a^ 0.16 ^b^ 0.15 ^b^
Stage III	34		
Stage IV	11		

Data are presented as the mean ± S.E.M., or number of cases (%). ^a^ Derived using the Mann–Whitney U test. ^b^ Derived using the chi-square test. AFS: American Fertility Society.

## Data Availability

The excel data used to support the findings of this study were supplied by Sung Hoon Kim under license, and requests for access to these data should be made to S.H.K.

## References

[1] Baldi A., Campioni M., Signorile P.G. (2008). Endometriosis: Pathogenesis, diagnosis, therapy and association with cancer. Oncol. Rep..

[2] Bulun S.E. (2009). Endometriosis. N. Engl. J. Med..

[3] Giudice L.C., Kao L.C. (2004). Endometriosis. Lancet.

[4] Haas D., Chvatal R., Reichert B., Renner S., Shebl O., Binder H., Wurm P., Oppelt P. (2012). Endometriosis: A premenopausal disease? Age pattern in 42,079 patients with endometriosis. Arch. Gynecol. Obstet..

[5] Schneider A., Touloupidis S., Papatsoris A.G., Triantafyllidis A., Kollias A., Schweppe K.W. (2006). Endometriosis of the urinary tract in women of reproductive age. Int. J. Urol..

[6] Practice Committee of the American Society for Reproductive Medicine (2004). Endometriosis and infertility. Fertil. Steril..

[7] Burney R.O., Giudice L.C. (2012). Pathogenesis and pathophysiology of endometriosis. Fertil. Steril..

[8] Attar E., Bulun S.E. (2006). Aromatase and other steroidogenic genes in endometriosis: Translational aspects. Hum. Reprod Update.

[9] Khan K.N., Kitajima M., Hiraki K., Fujishita A., Sekine I., Ishimaru T., Masuzaki H. (2008). Immunopathogenesis of pelvic endometriosis: Role of hepatocyte growth factor, macrophages and ovarian steroids. Am. J. Reprod. Immunol..

[10] Ramakrishna B.S. (2013). Role of the gut microbiota in human nutrition and metabolism. J. Gastroenterol. Hepatol..

[11] Schirbel A., Kessler S., Rieder F., West G., Rebert N., Asosingh K., McDonald C., Fiocchi C. (2013). Pro-angiogenic activity of TLRs and NLRs: A novel link between gut microbiota and intestinal angiogenesis. Gastroenterology.

[12] Zhang Y.J., Li S., Gan R.Y., Zhou T., Xu D.P., Li H.B. (2015). Impacts of gut bacteria on human health and diseases. Int. J. Mol. Sci..

[13] Costello M.E., Robinson P.C., Benham H., Brown M.A. (2015). The intestinal microbiome in human disease and how it relates to arthritis and spondyloarthritis. Best Pract. Res. Clin. Rheumatol..

[14] Khan K.N., Fujishita A., Hiraki K., Kitajima M., Nakashima M., Fushiki S., Kitawaki J. (2018). Bacterial contamination hypothesis: A new concept in endometriosis. Reprod. Med. Biol..

[15] Khan K.N., Fujishita A., Kitajima M., Hiraki K., Nakashima M., Masuzaki H. (2014). Intra-uterine microbial colonization and occurrence of endometritis in women with endometriosis. Hum. Reprod..

[16] Khan K.N., Fujishita A., Masumoto H., Muto H., Kitajima M., Masuzaki H., Kitawaki J. (2016). Molecular detection of intrauterine microbial colonization in women with endometriosis. Eur. J. Obstet. Gynecol. Reprod. Biol..

[17] Khan K.N., Kitajima M., Hiraki K., Yamaguchi N., Katamine S., Matsuyama T., Nakashima M., Fujishita A., Ishimaru T., Masuzaki H. (2010). Escherichia coli contamination of menstrual blood and effect of bacterial endotoxin on endometriosis. Fertil. Steril..

[18] Bailey M.T., Coe C.L. (2002). Endometriosis is associated with an altered profile of intestinal microflora in female rhesus monkeys. Hum. Reprod..

[19] Ata B., Yildiz S., Turkgeldi E., Brocal V.P., Dinleyici E.C., Moya A., Urman B. (2019). The Endobiota Study: Comparison of vaginal, cervical and gut microbiota between women with stage 3/4 endometriosis and healthy controls. Sci. Rep..

[20] Toyofuku M., Nomura N., Eberl L. (2019). Types and origins of bacterial membrane vesicles. Nat. Rev. Microbiol..

[21] Macia L., Nanan R., Hosseini-Beheshti E., Grau G.E. (2020). Host- and microbiota-derived extracellular vesicles, immune function, and disease development. Int. J. Mol. Sci..

[22] Brown L., Wolf J.M., Prados-Rosales R., Casadevall A. (2015). Through the wall: Extracellular vesicles in Gram-positive bacteria, mycobacteria and fungi. Nat. Rev. Microbiol..

[23] Choi Y., Park H., Park H.S., Kim Y.K. (2017). Extracellular vesicles, a key mediator to link environmental microbiota to airway immunity. Allergy Asthma Immunol. Res..

[24] Dagnelie M.A., Corvec S., Khammari A., Dréno B. (2020). Bacterial extracellular vesicles: A new way to decipher host-microbiota communications in inflammatory dermatoses. Exp. Dermatol..

[25] Choi Y., Kwon Y., Kim D.K., Jeon J., Jang S.C., Wang T., Ban M., Kim M.H., Jeon S.G., Kim M.S. (2015). Gut microbe-derived extracellular vesicles induce insulin resistance, thereby impairing glucose metabolism in skeletal muscle. Sci. Rep..

[26] Khalaj K., Miller J.E., Lingegowda H., Fazleabas A.T., Young S.L., Lessey B.A., Koti M., Tayade C. (2019). Extracellular vesicles from endometriosis patients are characterized by a unique miRNA-lncRNA signature. JCI Insight.

[27] Nazri H.M., Imran M., Fischer R., Heilig R., Manek S., Dragovic R.A., Kessler B.M., Zondervan K.T., Tapmeier T.T., Becker C.M. (2020). Characterization of exosomes in peritoneal fluid of endometriosis patients. Fertil. Steril..

[28] Chen C., Song X., Wei W., Zhong H., Dai J., Lan Z., Li F., Yu X., Feng Q., Wang Z. (2017). The microbiota continuum along the female reproductive tract and its relation to uterine-related diseases. Nat. Commun..

[29] Winters A.D., Romero R., Gervasi M.T., Gomez-Lopez N., Tran M.R., Flores V.G., Pacora P., Jung E., Hassan S.S., Hsu C.D. (2019). Does the endometrial cavity have a molecular microbial signature?. Sci. Rep..

[30] Wei W., Zhang X., Tang H., Zeng L., Wu R. (2020). Microbiota composition and distribution along the female reproductive tract of women with endometriosis. Ann. Clin. Microbiol. Antimicrob..

[31] Hernandes C., Silveira P., Rodrigues Sereia A.F., Christoff A.P., Mendes H., Valter de Oliveira L.F., Podgaec S. (2020). Microbiome profile of deep endometriosis patients: Comparison of vaginal fluid, endometrium and lesion. Diagnostics.

[32] Khan K.N., Kitajima M., Yamaguchi N., Fujishita A., Nakashima M., Ishimaru T., Masuzaki H. (2012). Role of prostaglandin E2 in bacterial growth in women with endometriosis. Hum. Reprod..

[33] (1997). Revised American Society for Reproductive Medicine classification of endometriosis: 1996. Fertil. Steril..

[34] Navas-Molina J.A., Peralta-Sánchez J.M., González A., McMurdie P.J., Vázquez-Baeza Y., Xu Z., Ursell L.K., Lauber C., Zhou H., Song S.J. (2013). Advancing our understanding of the human microbiome using QIIME. Methods Enzymol..

